# Integrating standardized whole genome sequence analysis with a global *Mycobacterium tuberculosis* antibiotic resistance knowledgebase

**DOI:** 10.1038/s41598-018-33731-1

**Published:** 2018-10-18

**Authors:** Matthew Ezewudo, Amanda Borens, Álvaro Chiner-Oms, Paolo Miotto, Leonid Chindelevitch, Angela M. Starks, Debra Hanna, Richard Liwski, Matteo Zignol, Christopher Gilpin, Stefan Niemann, Thomas Andreas Kohl, Robin M. Warren, Derrick Crook, Sebastien Gagneux, Sven Hoffner, Camilla Rodrigues, Iñaki Comas, David M. Engelthaler, David Alland, Leen Rigouts, Christoph Lange, Keertan Dheda, Rumina Hasan, Ruth McNerney, Daniela M. Cirillo, Marco Schito, Timothy C. Rodwell, James Posey

**Affiliations:** 1 Critical Path Institute, 1730 E River Rd., Tucson, AZ 85718 USA; 2 Joint unit Infection and Public Health FISABIO-CSISP/University of Valencia, Institute of integrative Systems Biology, Valencia, Spain; 3 Emerging Bacterial Pathogens Unit, IRCCS San Raffaele Scientific Institute, via Olgettina 58, 20132 Milano, Italy; 4 School of Computing Science, Simon Fraser University, 8888 University Ave, Burnaby, BC V5A 1S6 Canada; 5 Division of Tuberculosis Elimination, National Center for HIV/AIDS, Viral Hepatitis, STD, and TB Prevention, Centers for Disease Control and Prevention, 1600 Clifton Road MS F08, Atlanta, GA 30329 USA; 6 Global Tuberculosis Program, World Health Organization, Geneva, Switzerland; 7 German Center for Infection Research, Partner Site Borstel, Borstel, Germany; 8 Molecular and Experimental Mycobacteriology, Priority area Infections, Research Center Borstel, Borstel, Germany; 9 DST/NRF Centre of Excellence for Biomedical Tuberculosis Research/SAMRC Centre for Tuberculosis Research, Division of Molecular Biology and Human Genetics, Faculty of Medicine and Health Sciences, Stellenbosch University, Stellenbosch, South Africa; 10 Nuffield Department of Medicine, John Radcliffe Hospital, University of Oxford, Oxford, OX3 9DU United Kingdom; 11 Swiss Tropical and Public Health Institute, Basel, Switzerland; 12 Department of Public Health Sciences, Karolinska institute, Stockholm, Sweden; 13 Hinduja Hospital, Veer Savarkar Marg, Mahim, Mumbai, India; 14 Tuberculosis Genomics Unit, Biomedicine Institute of Valencia (IBV-CSIC), Street Jaime Roig 11. P.O., 4010 Valencia, Spain; 15 Translational Genomics Research Institute, 3051 W. Shamrell Blvd. Ste 106, Flagstaff, AZ 86005 USA; 16 Center for Emerging Pathogens, Rutgers-New Jersey Medical School, 185 South Orange Avenue, Newark, NJ 07103 USA; 17 Department of Biomedical Sciences, Institute of Tropical Medicine, Antwerp, Belgium; 18 Division of Clinical Infectious Diseases and German Center for Infection Research Tuberculosis Unit, Research Center Borstel, Borstel, Germany; 19 Lung Infection and Immunity Unit, Department of Medicine, Division of Pulmonology and UCT Lung Institute, University of Cape Town, Old Main Building, Groote Schuur Hospital, Observatory, Cape Town, South Africa; 20 Department of Pathology and Laboratory Medicine, Aga Khan University, Stadium Road, Karachi, Pakistan; 21 Department of Medicine, Division of Pulmonology, University of Cape Town, Groote Schuur Hospital, Cape Town, South Africa; 22 Department of Medicine, University of California, San Diego, CA USA

## Abstract

Drug-resistant tuberculosis poses a persistent public health threat. The ReSeqTB platform is a collaborative, curated knowledgebase, designed to standardize and aggregate global *Mycobacterium tuberculosis* complex (MTBC) variant data from whole genome sequencing (WGS) with phenotypic drug susceptibility testing (DST) and clinical data. We developed a unified analysis variant pipeline (UVP) (https://github.com/CPTR-ReSeqTB/UVP) to identify variants and assign lineage from MTBC sequence data. Stringent thresholds and quality control measures were incorporated in this open source tool. The pipeline was validated using a well-characterized dataset of 90 diverse MTBC isolates with conventional DST and DNA Sanger sequencing data. The UVP exhibited 98.9% agreement with the variants identified using Sanger sequencing and was 100% concordant with conventional methods of assigning lineage. We analyzed 4636 publicly available MTBC isolates in the ReSeqTB platform representing all seven major MTBC lineages. The variants detected have an above 94% accuracy of predicting drug based on the accompanying DST results in the platform. The aggregation of variants over time in the platform will establish confidence-graded mutations statistically associated with phenotypic drug resistance. These tools serve as critical reference standards for future molecular diagnostic assay developers, researchers, public health agencies and clinicians working towards the control of drug-resistant tuberculosis.

## Introduction

Tuberculosis (TB), caused by *Mycobacterium tuberculosis* complex (MTBC), was declared a global public health emergency in 1993^1^. However, TB remains a global public health threat and recently became the world’s number one cause of mortality from an infectious disease^2^. While there has been a gradual decline of TB incidence worldwide, the trend is not sufficient to end TB by 2035, a standing goal of the World Health Organization (WHO). The challenge is compounded by the worldwide spread of drug-resistant TB^3^.

Effective control of TB and a reduction in transmission of drug-resistant strains globally is dependent on the rapid detection and timely availability of drug susceptibility information for MTBC isolated from patients. These data are critical for effective antibiotic drug regimen selection and treatment monitoring which helps prevent transmission of drug resistant disease and achieve treatment success^4^. Culture-based, phenotypic drug susceptibility testing (DST), while considered the reference standard, requires specialized laboratory infrastructure, sophisticated biohazard sample transportation networks, and is technically difficult and time consuming. Additionally, established standards for current reference DST methods can be inconsistently implemented between laboratories and amongst assay methods for the drugs that are tested. The combination of slow MTBC replication and the challenges of growth-based phenotypic testing, results in delays of up to several weeks before clinicians receive laboratory results and can introduce a new drug combination for patients with increasingly complex resistance profiles^5,6^.

Detection of resistance–conferring genetic polymorphisms using molecular approaches such as polymerase chain reaction (PCR), DNA hybridization, targeted sequencing of specific genes, and whole genome sequencing (WGS) are promising rapid alternatives to phenotypic methods. Researchers initially focused their efforts on sequencing individual genomic loci known to confer antibiotic resistance in MTBC. Similarly, molecular typing methods such as IS6110-Restriction Fragment Length Polymorphism (RFLP), spoligotyping, and Mycobacteria interspersed repetitive units-Variable Number Tandem Repeat (MIRU-VNTR)^7–9^ have helped improve our understanding of MTBC epidemiology in terms of its genetic diversity, population structure and low-resolution transmission patterns. However, these approaches are relatively limited in their power to describe the full extent of natural variation in the MTBC population and cannot be used to develop a comprehensive picture of drug susceptibility determinants^10^. Sequencing of the first complete MTBC genome (H37Rv) in 1998^11^ empowered researchers to glean detailed information from whole genome sequences and address questions about how the genetic diversity within MTBC contributes to disease and drug resistance.

The advent of high-throughput next-generation sequencing (NGS) technologies have simplified and decreased the turn-around time for generating WGS data from MTBC clinical isolates. Researchers have used WGS analysis extensively to understand the molecular basis and host-ecosystem relationships in infectious diseases and microbiology. This has allowed advances in our understanding of epidemiology, pathogen evolution and virulence determinants to better conduct TB disease outbreak investigation and assess disease transmission networks^12,13^. With accessible benchtop sequencers and commercially available library preparation protocols, large quantities of data have been generated, resulting in a rapidly increasing number of publicly available raw genome sequences^14^.

NGS technologies appear poised to replace cumbersome culture-based DST for MTBC surveillance and, in some cases, patient management. However, several limitations prevent widespread implementation of WGS for clinical and public health uses. One key step in overcoming these hurdles is the adoption of user-friendly, validated bioinformatic tools to facilitate the use of WGS data by non-bioinformaticians. Additionally, we need a global collaborative effort to build and deploy a centralized knowledgebase for identifying the correlations between MTBC genotypes and phenotypic drug resistance to predict drug resistance to the arsenal of TB drugs available. Validated genomic variants curated in clinical knowledgebases will fill gaps in knowledge and eventually enable WGS-based methods to not only complement but also ultimately replace culture-based DST.

While the generation of DNA sequences is becoming simpler and less expensive, extracting, interpreting and communicating useful information in an easily understood format for clinical and public health audiences remains a significant challenge^2^ and few such bioinformatics resources exist^14,15^. Furthermore, a dearth of highly curated sequencing data and easy to use interpretation tools has slowed the progress of efforts for associating high-confidence mutations with resistance phenotypes or lineage groupings, which has negatively affected global confidence in the clinical relevance of some polymorphisms, discovered in NGS data.

There have been a number of TB-specific genome browsers and WGS analysis tools such as TGS-TB, TBProfiler, KvarQ, Mykrobe Predictor TB and PhyResSE developed within the past few years that are utilized for genotyping and drug resistance information^16–18^. Many of these tools have been helpful for correlating DST results with genotypic information from sequencing analysis^6,19^. However, to date, a comprehensive TB knowledgebase guided by a team of experts that presents standardized genomic data together with culture-based DST and clinical outcome metadata with linkages of observed polymorphisms to particular MTBC lineage groupings does not exist in the public domain.

ReSeqTB (https://platform.reseqtb.org) offers such a knowledgebase. Here, we describe a bioinformatics tool –the unified variant analysis pipeline (UVP) –, which is a suite of NGS analysis tools with defined quality metrics and a validated set of thresholds for standardizing the analysis of raw MTBC sequence data from Illumina sequencing platforms. As a result, raw sequencing files can be analyzed with an easy-to-use bioinformatics pipeline to provide standardized and reproducible high confidence genomic variant data. We also describe the ReSeqTB knowledgebase and its aggregation of variant reports from the UVP and accompanying metadata to produce a validated list of confidence-graded mutations associated with MTBC drug resistance phenotypes.

## Results

### UVP functionality

The UVP was developed through adoption of best practices and consensus thresholds and parameters for implementing NGS analysis tools agreed upon by the ReSeqTB panel of experts assembled globally from different fields relevant to the genomics approach of studying MTBC. The open source bioinformatics tools were implemented in the UVP to achieve two broad goals: identify high quality variants and assign MTBC lineage classification to each isolate analyzed.

The UVP consists of four broad sequential steps: 1) QC of the input sequence file, 2) mapping to the reference genome, 3) variant calling and annotation, and 4) MTBC lineage classification. The analysis pipeline accepts as input, WGS data in the form of Illumina short reads, which can be either paired-end or single-end reads. Prior to analyzing each sequence file, we checked the format of the file with FastQValidator^20^ to ensure adherence to the fastq format. As a pre-requisite for the analysis, the minimum acceptable average genome depth of coverage was 10×. Briefly, each isolate WGS reads is assessed for MTBC species specificity using Kraken^21^, and 90% of the reads had to map to MTBC for acceptance. The accepted fastq files were assessed for quality and trimmed using FastQC and Prinseq^22^. ensuring an average read quality of Q20, and then mapped against the *Mycobacterium tuberculosis* reference genome H37Rv, GenBank accession no. NC_000962.3 using BWA-MEM^23^. To reduce the number of false positive variants, duplicates were removed from the BAM files using PICARD tools^24^. We performed base call re-calibration and re-alignment around small Indels in the genome with the Genome Analysis Toolkit (GATK)^25^. GATK and Samtools^26^ are used to call both Single Nucleotide Polymorphisms (SNPs) and Indels, and the functional annotation tool SnpEff^27^ is used to annotate the output variant file. We inferred lineage classification for each isolate by cross-referencing to a set of 62 informative SNPs outlined by Coll *et al*.^28^. The criteria set for identifying SNPs and small Indels includes: Q20 minimum base call quality score, a minimum Q20 mapping quality score, presence of variants on both strands for paired reads, maximum of 3 SNPs within a span 10 bp and a minimum coverage depth of 10X for each identified variant. The UVP also checks for multiple mutations in the ribosomal genes of MTBC (*rrs* and *rrl*). These regions are normally conserved^29^ and samples with more than five mutations within these regions are flagged as mixtures or contamination.

We implemented the UVP with custom Python programing scripts at different points of the analysis to facilitate processing and data flow. The primary scripts included *lineage_parser*.*py* that parses the variation file to infer MTBC lineage assignment, *parse_annotation*.*py* that formats the SnpEff annotated variation file into a more user-friendly text format and a suite of custom scripts that generate coverage information for all genomic loci (*coverage_estimator*.*py*, *resis_parser*.*py and del_parser*.*py*). This last set of scripts — unique to the UVP — dynamically queried the genome coverage reports generated across the entire genome to infer loci that have complete or partial deletions.

The UVP analysis generated three key result files in addition to a number of reports from each of the QC steps. The first is an annotation file that lists the variant positions across the isolate genome including the quality scores, coverage and annotation details for each position. The variants in this annotation file feeds the ReSeqTB platform, and is aggregated with phenotypic DST data from same isolates to make predictions on antibiotic resistance. Next is the lineage report file that infers the MTBC lineage assignment for the isolate out of the possible seven major MTBC lineages defined in the work by Coll *et al*.^28^. Finally, the genome coverage report lists the average depth and width of coverage across all the loci in the isolate genome.

The UVP source code and details on the pipeline tools and thresholds set for these tools are available at https://github.com/CPTR-ReSeqTB/UVP.

### UVP Validation Results

#### MTBC lineage assignment validation

Our analyses demonstrated 100% concordance in the assignment of lineages between the conventional genotyping methods and the UVP for the 79 strains where conventional methods could infer a specific lineage. Both the UVP and the PhyResSE analysis tool (used as a comparator) inferred all the 11 strains that had no lineage classification using conventional genotyping methods to belong to the Euro-American lineage. The rest of the lineage classification analysis showed 100% concordance between PhyResSE and the UVP on the assignment of lineages for all 90 strains analyzed (see Supplementary Data 1).

#### SNPs and Indels detection validation

In the test for concordance of variant calls between Sanger sequencing analysis and the UVP there was 100% concordance in resistance associated SNPs detected across two resistance loci (*rpsL* and *katG*) for all 90 strains. For the remaining loci of interest, there was 98.9% agreement. The UVP identified the discordant resistance associated SNPs in each case. This instance, also observed in PhyResSE analysis of the same data, suggests hetero-resistant samples, which are a mixture of wild type and variant alleles that Sanger sequencing was not able to detect^30^ (Table 1). We also observed 100% concordance in the SNP calling results between PhyResSE and the UVP (Table 1).The evaluation work on the same dataset, which showed that PhyResSE compares favorably to other NGS data analysis tools^18^ offers confidence against possible systematic errors in variant detection by both the UVP and PhyResSE. For validation of detection of short Indels, we used additional isolates drawn from previously analyzed WGS analyses to assess genomic heterogeneity in clinical MTBC isolates^31^ to establish the utility of the UVP. We compared the results of the UVP analysis of these isolates to that of the analysis of the validated data, and for each deletion there was a 100% agreement on the genomic location and size of the deletions (Table 2).

**Table 1:** Evaluation of Unified variant pipeline SNP calling versus SNP detection using Sanger sequencing and PhyResSE analysis pipeline.

Resistance gene	# of SNPs detected by Sanger sequencing	# of SNPs detected by UVP	# of discordant SNPs between UVP and Sanger sequencing	% Concordance between UVP and Sanger sequencing	# of SNPs detected by PhyResSE	% Concordance between UVP and PhyResSE
*rpsL*	19	19	0	100	19	100
*gidB*	66	67	1	98.9	67	100
*katG*	25	25	0	100	25	100
*rpoB*	19	20	1	98.9	20	100
*embB*	16	17	1	98.9	17	100
*pncA*	11	12	1	98.9	12	100

**Table 2:** Comparisons of Indels calls by UVP and validated variants previously shown to be present in given isolates.

Sample ID	PubMed ID	Affected gene	H37Rv reference genomic position	Indel	UVP inferred Indel	Lineage information	*tap* gene*(Rv1258c*) frameshift mutation
ERR502929	27021327	dfrA, thyA	3073130–3074471	complete deletion	complete deletion	2.2.1	present
ERR751361	27021327	Wt	3073130–3074471	no deletion	no deletion	2.2.1	present
ERR751453	27021327	dfrA, thyA	3073130–3074471	complete deletion	complete deletion	2.2.1	present
ERR751483	27021327	Wt	3073130–3074471	no deletion	no deletion	2.2.1	present
ERR775373	27021327	dfrA, thyA	3073130–3074471	complete deletion	complete deletion	2.2.1	present
ERR779910	27021327	dfrA, thyA	3073130–3074471	complete deletion	complete deletion	2.2.1	present
SRR2333215	27021327	dfrA, thyA	3073130–3074471	complete deletion	complete deletion	2.2.2	absent
SRR1952721	25977398	pncA	2288681–2289241	complete deletion	complete deletion	2.2.1	present
SRR1948177	25977398	pncA	2288681–2289241	complete deletion	complete deletion	2.2.1	present
R721_C13	26496891	atsD	756970	C > CG	C > CG	2.2.1.1	present
R721_C13	26496891	regX3	581317	G > GCG	G > GCG	2.2.1.1	present
R966_C5	26496891	Rv3861	4338065	AC > A	AC > A	2.2.1	present
R160_C13	26496891	adhD	3452173	CG > CGCCAT	CG > CGCCATG	4.1.1.3	absent
R376_C11	26496891	Rv3256c	3636924	ACC > AC	ACC > AC	4.1.2.1	absent
R912_C11	26496891	yrbE4A	3920814	GA > G	GAA > GA	1.2.2	absent
R965_C1	26496891	adhA	2110232	A > AG	AG > AGG	2.2.1	present
R458_C3	26496891	espR	4323810	GC > G	GC > G	4.1.2	absent
R641_C1	26496891	Rv2375	2655550	A > AT	AT > ATT	2.2.1.1	present

#### Large structural variations and gene deletion validation

Analysis of the simulated WGS data with varying length Indels in the third dataset demonstrated the pipeline’s variant calling tool could identify long Indels (up to 50 bp) at their accurate genomic positions (Supplementary Table S1). From our simulations, we deduced that neither the GATK variant caller nor Samtools detect Indels greater than about 50 bp long for the simulated sequences, using the thresholds and settings implemented in the pipeline. This result expands on previous findings that most NGS data analysis tools accurately identify short (<10 bp) insertions and deletions^32^, suggesting that biologically relevant structural variations of only up to half of the average read lengths in MTBC genomes can be accurately inferred by UVP just using the GATK or Samtools variant callers. We therefore implemented a custom script within the UVP to capture such large deletions that are more than half the length of the average sequencing read for each isolate. To test this, we reanalyzed MTBC isolates (ERR502929, ERR751361, ERR751453, ERR751483, ERR775373, ERR779910, SRR2333215, SRR1952721 and SRR1948177) previously shown to have complete gene deletions across several loci of interest (*i*.*e*. *pncA*, *dfrA*, and *thyA)*^33,34^. In addition, we searched for the *tap* gene deletion previously shown to be present in most MTBC strains classified into the Beijing lineage^35^ in the output of UVP analysis of the isolates. The results from the analysis using our custom script that infers large deletions across the genome, showed that the respective *pncA*, *thyA*, and *dfrA* loci were absent in all the strains except for the wild type isolates (Table 2). The *tap* gene deletion that leads to a frameshift mutation was present in all the strains of East Asian lineage (Lineage 2) analyzed except for SRR2333215, which belongs to a different East Asian sub-lineage (2.2.2). For isolates belonging to other MTBC lineages, this variant was absent as expected based on previous studies (Table 2).

#### Antibiotic resistance prediction

The ReSeqTB platform independently calculates the Likelihood ratio test statistic (LR+) as previously described^36^ for all variants found in loci of interest (see Supplementary Data 1) thought to be associated with resistance to corresponding antibiotics. The LR+ test offers a robust approach to assigning the strength of association between variants and their corresponding drug resistance phenotypes. Mutations that have high LR+ (>10) values for the test of association to drug resistance in this context would offer strong confidence of association to drug resistance. Currently, applying the LR+ test, there are 53 such mutations on the platform (platform.reseqtb.org) with a value >10 and classed as high confidence mutations. There is corroboration that these 53 mutations are associated with antibiotic resistance in MTBC through work done over the years by different researchers in this field^37–39^, but the numbers of such mutations is limited by the underlying dataset from data currently in the ReSeqTB platform. To address this limitation for the purposes of our analysis, we created a comprehensive list of high confidence mutations, by adding the 53 mutations to the list of high confidence mutations described in the systematic review analysis work done by Miotto *et al*.^36^ (Supplementary Data 2). Most of the initial 53 mutations (except the *fabG1* synonymous mutation) with LR+ values >10 from the platform are also present in the list. The comprehensive list was then used to test for the accuracy of predicting antibiotic resistance reported in the phenotypic data housed in the platform and captured in our dataset.

**Figure 1:**
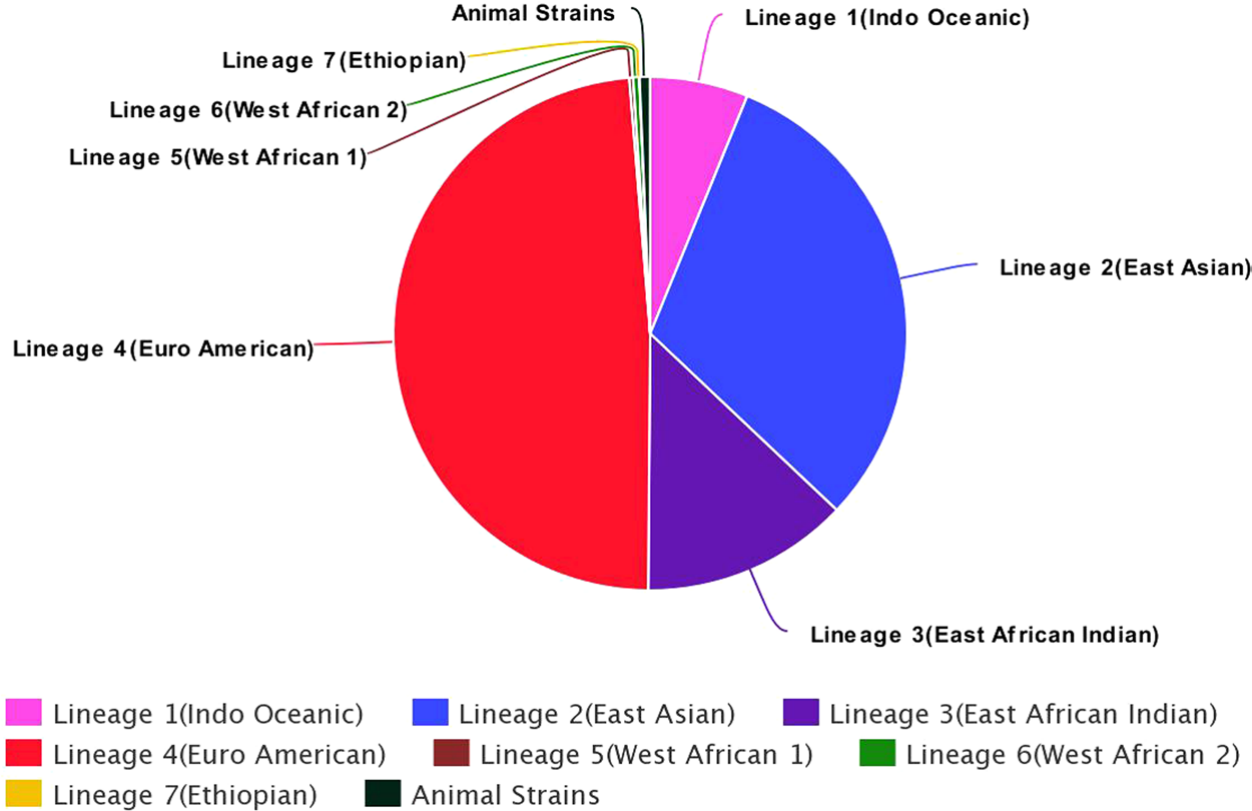
Distribution of isolates on ReSeqTB platform among the seven major lineages of the *Mycobacterium tuberculosis* complex (MTBC). This schematic shows the proportional representation of the major MTBC lineages in our dataset. The Euro American lineage has the most representation, but every other major lineage including Lineage 7 and animal strains are represented in the dataset.

Our analysis to test for the predictive value of variants in the comprehensive list was specifically targeted on 829/4636 isolates in the dataset (see Supplementary Data 3) with known phenotypic test results for multiple drugs using MGIT 960 and drawn from all major MTBC lineages (see Fig. 1). The analysis, using phenotypic DST as the standard, showed that high confidence mutations in loci associated with resistance to the first line drugs isoniazid and rifampicin had 94.9% and 97.5% accuracy (measured in each instance as the ratio of true calls to the total number of calls for each drug), respectively, for predicting resistance to these drugs. High confidence mutations associated with resistance to fluoroquinolones in the dataset, had 99.1% and 97.5% accuracy for predicting resistance for levofloxacin and ofloxacin, respectively. For the first line drugs, we found several variants present in isolates that are phenotypically resistant but lack any of the high confidence mutations from our list (Supplementary Data 2). Table 3 provides details on the accuracy, sensitivity and specificity (including their associated binomial 95% confidence intervals) results for the MGIT method. Further details on the distribution of high confidence mutations for the first line antibiotic drugs in our dataset is provided in Supplementary data 2. The accuracy values for associating drug resistance to high confidence mutations with results from DST on solid media and those without a specified method (this affects 3807/4636 isolates in the dataset which make up 82% of the phenotypic testing results), were not included in our major analysis. In the former case, we lacked sufficient sample size for DST in solid media for our analysis, and in the latter case, the lack of specific DST methods introduced some level of ambiguity to the results when pooled. Separately, we performed the resistance prediction analysis on these excluded 82% of the dataset, in Supplementary Data 4, and showed sensitivity values as much as 10% lower (0.81–0.85 for solid media testing; 0.85–0.88 for DST with no stated method) when compared to results using MGIT DST results alone. In addition, we listed putative drug resistance mutations within the loci of interest that could explain this relative drop in sensitivity for these excluded data. We did not include test results for ethambutol, pyrazinamide, moxifloxacin and the second-line injectable agents because they lack sufficient sample size to make clear inferences on the accuracy measure although they suggest the same trends.

**Table 3:** Sensitivity, specificity and accuracy values of drug resistance prediction by high confidence mutations inferred to be associated with antibiotic resistance in isolates within the data set with DST results from the BACTEC MGIT 960 Mycobacteria liquid culture method.

Drug	TP	FP	TN	FN	Sensitivity	95%CI Low	95% CI High	Specificity	95%CI Low	95%CI High	Accuracy (%)
Isoniazid	321	1	463	41	0.887	0.848	0.917	0.997	0.986	0.999	94.9
Levofloxacin	23	10	313	2	0.92	0.725	0.986	0.969	0.942	0.984	96.6
Ofloxacin	35	1	312	2	0.946	0.805	0.991	0.997	0.978	0.999	99.1
Rifampicin	275	2	533	19	0.935	0.899	0.96	0.996	0.985	0.999	97.5

## Discussion

The UVP described herein, which processes raw NGS data for submission to the ReSeqTB knowledgebase, was developed by applying best practices and quality checks. These protocols were put in place by a global consortium of experts within the TB and genomics research fields that work to interpret data driven results from bioinformatic tools and computational databases analytics, drawing from a consensus on prior knowledge and experience in the field. This collaboration, which is a unique feature of the UVP and the ReSeqTB, could serve as a template for future collaborations around scientific endeavors that are of public health relevance. The UVP currently accepts WGS data from Illumina platforms and future advancements will include added capacity to process WGS from a variety of other NGS platforms to add to the knowledge base. In addition, future efforts will include the capacity to analyze sequence data from targeted sequencing approaches, which will enable capture of sequencing data from clinical samples. This is critical to ensure inclusion of data from settings where a culture-based approach is not routinely used. An online user- interactive version of this publicly available tool is under development and information on this will be made available on the public GitHub site once it is accessible.

In addition and unique to the UVP, algorithms implemented in the pipeline enable easy accounting for loci in the MTBC genome where there are large deletions. Typically, Indels longer than the sequence read length from NGS platforms are not accounted for using regular bioinformatics analysis tools. The UVP analysis captures such records and is important for loci where large deletions could underlie antibiotic resistance phenotypes. If not properly identified, these deletions could confound attempts to establish novel and comprehensive associations with resistance phenotypes. There are two limitations for the UVP using this approach. First, the pipeline does not account for large insertions beyond those detected by the variant calling tools in the pipeline. Additionally, the UVP cannot yet reliably identify heterogeneous Indels (mixture of wild type and Indels at same location) in clinical isolates that have minor sub-populations. This ties to the broader challenge of developing algorithms with robust hetero-resistance detection in general. Such algorithms should apply rigorous thresholds on allele frequencies and coverage depth at loci of interest in order to produce accurate predictions of phenotype from sequence information. These improvements will also help refine all antibiotic resistance prediction and phylogenetic lineage classification for isolates with and without sub-populations. Moreover, the clinical relevance of such minor populations will need to be evaluated for each drug taking into account the totality of resistance information available and the treatment regimen employed.

The UVP compared to other NGS analysis tools, uniquely applies a rigorous approach to identify mixed or contaminated samples, going beyond just the species specificity checks implemented in the Kraken tool, but also looking for patterns of variations within conserved ribosomal genes of MTBC strains that will suggest presence of multiple species within a given sample. The consensus approach described here of first establishing thresholds and criteria for the various NGS analytical tools implemented in a given analysis pipeline, and then validating against well-curated data, could point the way towards standardizing bioinformatics tools and approaches for analyzing NGS data in the future. However, there is still the need for an expanded validation dataset drawn from primary samples to answer specific questions on the presence and impact of closely related species such as non-tuberculosis mycobacteria (NTMs) on the analyses.

While there are a number of other MTBC WGS analysis pipelines or tools such as TB-Profiler, GenTB, CASTB, KvarQ that employ a number of approaches to infer variants and predict drug resistance in isolates. Our intent on settling on PhyResSE as the tool to perform our validation analysis comparisons is because of the fact that this tool applies a similar reference genome guided approach of identifying genetic variants like the UVP, and has also been compared with a number of other WGS analysis tool in a recent publication^18^, making it a good candidate for our validation comparisons.

The ReSeqTB knowledgebase includes an investigational database that standardizes and aggregates quality validated data submitted to the platform by contributors. This contributed data includes genomic sequence data, phenotypic DST results and clinical outcome data when available. In the long term, other potentially useful data types including surveillance data and clinical trial data should be included on the platform to provide broader context and information to different categories of users. The combination of the UVP and the ReSeqTB data platform uniquely enhances the capability to both confirm underlying antibiotic resistance phenotypes and potentially predict novel resistance variants. The antibiotic resistance prediction analysis we conducted on a set of 829 isolates serves to point to this capability, but it also demonstrated some inherent limitations in our approach. One such limitation is the number of publicly available data on the platform. We performed the resistance prediction accuracy analysis on only a subset of the over 7800 contributed datasets currently aggregated on the platform. This issue will be assuaged as more curated genetically and phenotypically diverse and publicly available isolate data populate the ReSeqTB database. Another limitation tied to the dataset on the platform is the limited number of isolates whose DST results specifies the actual method used for the testing. We settled on isolates with complete information on the DST methods for this proof of concept analysis, which lead to the exclusion of more than 80% of the publicly available dataset on the platform at the time of the analysis, from our major analysis. As more data accumulate on the platform, we will perform validation-type analysis on the subset of data without test methods attached to their DST results, to assess the accuracy values from those. The goal will be to integrate the entire dataset pool for future resistance-prediction analysis. In our current resistance prediction analysis on isolates tested with the MGIT DST method, we detected several variants that could account for the drop in accuracy for the association of drug resistance to the first line drugs. For isoniazid, of the 44 false negatives in the test for sensitivity, we highlighted 38 mutations, but also identified frameshift mutations and Indels within the *inhA*, *fabG1*, *katG* and *ahpC* upstream loci such as the Leu203Leu *fabG1* mutation, the −17G > T *fabG1* upstream mutation and *katG* Ser315 mutations (other than Ser315Thr) in those isolates, which could explain this discrepancy. These mutations are known to confer resistance to isoniazid but are not yet considered as high confidence in our dataset because they are not yet at an appreciable frequency in the database. Similarly for rifampin resistance we highlighted an additional 12 mutations within the *rpoB* loci which could explain some of the 19 false negatives observed in the sensitivity calculations. A number of these mutations are codon 445 *rpoB* mutations previously described in the literature to be associated with rifampin resistance^40,41^. Some of the other mutations are novel and would be candidates for functional genomic studies to assess their association with drug resistance. The details on the frequency and distribution of these mutations are in Supplementary Data 2. In addition, it has been well documented that liquid media DST methods often fail to identify some resistance to rifampicin^36^ and could account for some of the drop in our accuracy measure for rifampicin resistance prediction. For the supplementary work we did on isolates with DST results that either have no stated methods or were performed on solid media, the results point to similar explanation for the drop in sensitivity overall, and compared to the MGIT DST results. For isoniazid resistance, the false negatives in the sensitivity calculations for the solid media method and the no stated DST method were 33 and 147 respectively, and there were 31 and 66 mutations respectively in the isolates tested that could explain some of the drop insensitivity, given they are known to underlie resistance. For rifampin, there were 65 mutations which could explain part of the 128 false negatives observed for isolates where there is no stated method for the DST results, and a combination of 33 mutations which have been shown to confer resistance to rifampicin that could explain the 30 false negatives observed for the subset tested using solid media. Details of the results on this supplementary work can be found in Supplementary Data 4. These mutations, although previously shown to be associated to the respective drug resistance phenotypes, were not classed as high confidence in the ReSeqTB database due to the size limitations of our dataset. But as more data are aggregated in the platform, the frequencies of such mutations will increase, which will upgrade them to high confidence status and help refine the accuracy and sensitivity of our approach.

Similarly, the approach of using the LR+ test statistic to infer associations between mutations and resistance phenotypes for any dataset serves to give a broad picture of mutations or variants associated with antibiotic resistance. The statistic by itself is limited in explaining instances where mutations could be of low frequency within the sampled population and may not rise to appreciable frequency for the LR+ calculations (e.g. *pncA* loci of interest for pyrazinamide). The panel of experts from the TB research community were consulted to consider these limitations in assessing the ability of mutations to predict resistance to anti-TB medicines and as a future step will be working to develop a holistic grading system for mutations. In addition to the LR+ test statistic of association to phenotypic resistance, this grading system will also take into consideration many other factors including homoplasy to exclude lineage markers coincidentally found in drug-resistant strains. Metadata in the ReSeqTB platform including MICs, clinical outcomes, and functional analysis on the mutation in question could also be considered in assessing the role that a polymorphism has in drug resistance. This will ultimately result in a dynamic knowledgebase and consensus driven approach to assess the impact of resistance variants as suggested by the prediction analysis as data aggregates in the platform.

There is an increasing need to improve the process of routine identification and DST for TB and other microbial pathogens in the clinical setting. The adoption of bacterial WGS by clinicians and public health officials could open that door, if best practices are implemented in both identifying variants within isolate genomes and rendering confidence grading on the interpretation of such variants to drug resistance or other phenotypes of interest. The suite of bioinformatics and statistical analysis tools offered by ReSeqTB could prove invaluable to laboratories that can generate NGS data but lack the expertise and resources to analyze the increasing amount of high- throughput data coming from their laboratories. The approach of the UVP integrated into the ReSeqTB platform could indeed serve as a model in the future, where developers and researchers would increasingly rely on high confidence mutations, identified from high quality curated data to systematically show association with phenotypes of interests via a robust and knowledge-driven process.

## Methods

### Validation of UVP

We evaluated the UVP for accuracy of variant calling and lineage assignment by evaluating UVP performance on a highly curated data set with well-established genotypes. This analysis included three different sample sets:

*(i)* 90 MTBC strains drawn from a well-characterized strain collection from Sierra Leone in West Africa^30,42,43^. This data set consisted of 43 strains that were phenotypically resistant to all antibiotics tested, and 47 strains that were resistant to either one or a combination of the antibiotic drugs used for the DST testing (details in Supplementary Data 1). We inferred antibiotic resistance phenotypes to five anti-tuberculosis drugs: rifampicin (RIF), isoniazid (INH), streptomycin (SM), ethambutol (EMB), and pyrazinamide (PZA). Additionally, the results of the analysis were compared to results from a similar analysis performed using the well-documented NGS analysis pipeline PhyResSE^30^. PhyResSE has also independently been compared to four other MTBC WGS analysis software and shown to be optimal in terms of functionality, lineage assignment and variant calling amongst the compared tools^18^. The data set included previously described DST results, conventional sequencing data for six TB drug resistance-associated loci (*rpsL*, *gidB*, *katG*, *rpoB*, *embB*, and *pncA*) and WGS reads of all the strains in the data set^30^. We retrieved the WGS data from the EMBL-EBI-ENA sequence read archive with the project ID: PRJEB7727. The data also contained phylogenetic strain classifications obtained through conventional genotyping methods as well as from PhyResSE web tool analysis. For this first sample set, we performed two sets of validation analyses. The first analysis established the level of agreement on the classification of the strains using conventional genotyping methods (spoligotyping, 24-locus MIRU-VNTR, and IS6110 DNA fingerprinting) versus the UVP analysis. We performed similar lineage classification analyses based on results from both the UVP and the PhyResSE application. For the second analysis, we checked and confirmed the agreement on the variants identified in the regions of interest within all 90 strains using the UVP against Sanger sequencing variant detection.

*(ii)* We utilized a second sample set that comprised a number of WGS data sets previously described in the MTBC literature^31,33,34^ to evaluate how the pipeline handles both small insertions and deletions (Indels). These curated data had deletions within loci of interest mapped against the H37Rv reference genome that were validated using Sanger sequencing. WGS in this data set are available in the EMBL-EBI-ENA sequence read archive with the project ID: PRJEB7727^31^.

*(iii)* A data set comprised of simulated WGS reads generated using the ART tool^44^ was used to establish the sensitivity of the analysis suite for calling Indels in MTBC WGS data sets. Indels are problematic features when analyzing NGS data and long Indels that span the length of sequencing reads could prove particularly difficult to identify using current bioinformatics analysis tools^45^. We described the simulated sequences in Supplementary Table 1, demonstrating deletions with sizes ranging from one base pair (bp) to 100 bp in length. Also additional WGS available in the NCBI sequence read archive accession numbers ERR502929, ERR751361, ERR751453, ERR751483, ERR775373, ERR779910, SRR233215, SRR1952721 and SRR1948177, were used to assess how UVP handles large deletions or complete deletions of genes within loci of interest.

### Simulation of Illumina WGS reads

Synthetic fastq files were generated using a custom Perl script, *fasta_insert_deletions*.*pl* (https://gitlab.com/tbgenomicsunit/Publications_resources) and the ART tool^44^ to simulate insertions and deletions (Indels) of varying lengths. The Perl script accepts as input, a reference genome, an annotation file and a target gene. The reference genomes are complete fasta format genome files downloaded from NCBI repositories. The Perl script creates a set of new genomes based on the reference. Each simulated genome has randomly assigned insertions and deletions ranging from one to 100 bp within the target gene supplied to the script. These simulated genomes are used as an input by the ART tool to generate the fastq files. We created paired-end fastq files, simulating the Illumina HiSeq2500 technology, with a read coverage of 50x and read length of 150 bp. We used the available MTBC genome in the tool *Mycobacterium africanum* genome (GM041182, NC_015758.1) as the reference, and a number of MTBC genes of interest as target loci.

### Antibiotic resistance prediction using aggregated data in the ReSeqTB platform

The panel of drugs and corresponding loci of interest used in the analysis included isoniazid *(inhA*, *fabG1* and *katG*), rifampicin (*rpoB*), levofloxacin, ofloxacin, moxifloxacin (*gyrA* and *gyrB*), kanamycin (*rrs*, *eis* and *whiB7*), capreomycin (*rrs* and *tlyA*) and amikacin (*rrs*).

Phenotypic testing was performed on the 4636 isolates in the dataset submitted to the ReSeqTB platform through December 2017, and provided either a categorical result or Minimum Inhibitory concentration (MIC) using a variety of DST methods by the data contributors to the ReSeqTB knowledgebase. About 80% of the phenotypic test results were categorical DST without details on the specific methods used. Of the remaining DST results where a method was provided, 67% had DST performed using the liquid medium BACTEC Mycobacterial Growth Indicator Tube 960 (MGIT). Details of the phenotypic testing methods results are shown in Supplementary Data 2 and Supplementary Data 3.

Likelihood Ratio (LR+) is calculated using the formulas below:

$$\begin{array}{ll}{\rm{a}}. & {\rm{Sensitivity}}={\rm{TP}}/({\rm{TP}}+{\rm{FN}})\\ {\rm{b}}. & {\rm{Specificity}}={\rm{TN}}/({\rm{TN}}+{\rm{FP}})\\ {\rm{c}}. & {\rm{PPV}}={\rm{TP}}/({\rm{TP}}+{\rm{FP}})\\ {\rm{d}}. & {\rm{NPV}}={\rm{TN}}/({\rm{TN}}+{\rm{FN}})\\ {\rm{e}}. & {\rm{LR}}\,+={\rm{Sensitivity}}/1-{\rm{Specificity}}\end{array}$$

where TP means true positive, FN means false negative, PPV means positive predictive value and NPV means negative predictive value.

## Electronic supplementary material

Supplementary Information

Supplementary Data 1

Supplementary Data 2

Supplementary Data 3

Supplementary Data 4

## Data Availability

Sequence reads for isolates used in this analysis are available publicly in the NCBI sequence read archives under project ids: PRJNA282721, PRJNA200335, PRJNA244659, PRJNA240330 and PRJEB7798 and deposited as publicly available data on the ReSeqTB data platform (platform.reseqtb.org).
