# Author Correction: Integrating standardized whole genome sequence analysis with a global *Mycobacterium tuberculosis* antibiotic resistance knowledgebase

**DOI:** 10.1038/s41598-020-58955-y

**Published:** 2020-02-21

**Authors:** Matthew Ezewudo, Amanda Borens, Álvaro Chiner-Oms, Paolo Miotto, Leonid Chindelevitch, Angela M. Starks, Debra Hanna, Richard Liwski, Matteo Zignol, Christopher Gilpin, Stefan Niemann, Thomas Andreas Kohl, Robin M. Warren, Derrick Crook, Sebastien Gagneux, Sven Hoffner, Camilla Rodrigues, Iñaki Comas, David M. Engelthaler, David Alland, Leen Rigouts, Christoph Lange, Keertan Dheda, Rumina Hasan, Ruth McNerney, Daniela M. Cirillo, Marco Schito, Timothy C. Rodwell, James Posey

**Affiliations:** 1 Critical Path Institute, 1730 E River Rd., Tucson, AZ 85718 USA; 2 Joint unit Infection and Public Health FISABIO-CSISP/University of Valencia, Institute of integrative Systems Biology, Valencia, Spain; 3 Emerging Bacterial Pathogens Unit, IRCCS San Raffaele Scientific Institute, via Olgettina 58, 20132 Milano, Italy; 4 School of Computing Science, Simon Fraser University, 8888 University Ave, Burnaby, BC V5A 1S6 Canada; 5 Division of Tuberculosis Elimination, National Center for HIV/AIDS, Viral Hepatitis, STD, and TB Prevention, Centers for Disease Control and Prevention, 1600 Clifton Road MS F08, Atlanta, GA 30329 USA; 6 Global Tuberculosis Program, World Health Organization, Geneva, Switzerland; 7 German Center for Infection Research, Partner Site Borstel, Borstel, Germany; 8 Molecular and Experimental Mycobacteriology, Priority area Infections, Research Center Borstel, Borstel, Germany; 9 DST/NRF Centre of Excellence for Biomedical Tuberculosis Research/SAMRC Centre for Tuberculosis Research, Division of Molecular Biology and Human Genetics, Faculty of Medicine and Health Sciences, Stellenbosch University, Stellenbosch, South Africa; 10 Nuffield Department of Medicine, John Radcliffe Hospital, University of Oxford, Oxford, OX3 9DU United Kingdom; 11 Swiss Tropical and Public Health Institute, Basel, Switzerland; 12 Department of Public Health Sciences, Karolinska institute, Stockholm, Sweden; 13 Hinduja Hospital, Veer Savarkar Marg, Mahim, Mumbai, India; 14 Tuberculosis Genomics Unit, Biomedicine Institute of Valencia (IBV-CSIC), Street Jaime Roig 11. P.O., 4010 Valencia, Spain; 15 Translational Genomics Research Institute, 3051 W. Shamrell Blvd. Ste 106, Flagstaff, AZ 86005 USA; 16 Center for Emerging Pathogens, Rutgers-New Jersey Medical School, 185 South Orange Avenue, Newark, NJ 07103 USA; 17 Department of Biomedical Sciences, Institute of Tropical Medicine, Antwerp, Belgium; 18 Division of Clinical Infectious Diseases and German Center for Infection Research Tuberculosis Unit, Research Center Borstel, Borstel, Germany; 19 Lung Infection and Immunity Unit, Department of Medicine, Division of Pulmonology and UCT Lung Institute, University of Cape Town, Old Main Building, Groote Schuur Hospital, Observatory, Cape Town, South Africa; 20 Department of Pathology and Laboratory Medicine, Aga Khan University, Stadium Road, Karachi, Pakistan; 21 Department of Medicine, Division of Pulmonology, University of Cape Town, Groote Schuur Hospital, Cape Town, South Africa; 22 Department of Medicine, University of California, San Diego, CA USA; 23 The Foundation for Innovative New Diagnostics, Geneva, Switzerland

Correction to: *Scientific Reports* 10.1038/s41598-018-33731-1, published online 18 October 2018

The original version of this Article omitted an affiliation for Timothy C. Rodwell. The correct affiliations for Timothy C. Rodwell are listed below:

Department of Medicine, University of California, San Diego, CA, USA

The Foundation for Innovative New Diagnostics, Geneva, Switzerland.

This has now been corrected in the HTML and PDF versions of this Article.

