# Supplementary material for: Integrating standardized whole genome sequence analysis with a global Mycobacterium tuberculosis antibiotic resistance knowledgebase
Source: Sci Rep. 2018 Oct 18;8:15382. doi: 10.1038/s41598-018-33731-1 (PMC6194142; doi:10.1038/s41598-018-33731-1)
Supplement: Supplementary file 1 — Supplementary Information [file 41598_2018_33731_MOESM1_ESM.docx]

**Integrating standardized whole genome sequence analysis with a global *Mycobacterium tuberculosis* antibiotic resistance knowledgebase**

**Authors:**

Matthew Ezewudo, Amanda Borens, Álvaro Chiner-Oms, Paolo Miotto, Leonid Chindelevitch, Angela M. Starks, Debra Hanna, Richard Liwski, Matteo Zignol, Christopher Gilpin, Stefan Niemann, Thomas Andreas Kohl, Robin M. Warren, Derrick Crook, Sebastien Gagneux, Sven Hoffner, Camilla Rodrigues, Iñaki Comas, David M. Engelthaler, David Alland, Leen Rigouts, Christoph Lange, Keertan Dheda, Rumina Hasan, Ruth McNerney, Daniela M. Cirillo, Marco Schito, Timothy C. Rodwell, James Posey

**Supplementary Information**

**1. Unified variant pipeline (UVP)**

The UVP incorporates a suite of the most current bioinformatics analysis tools, written in python scripting language.

In broad outline, there are four major steps implemented in the pipeline:

1. Input data validation & QC
2. Sequence reads mapping & refinement
3. Variant calling
4. Functional annotation & lineage analysis

At least one or more bioinformatics tools are employed for each of these steps, to perform analysis on the data to generate an output file which is subsequently passed on to the next analysis step of the pipeline.

**1.1 Input data validation & QC**

Current Input data for the pipeline will be in fastq format from illumina NGS technology platform. There is a need to ascertain that data accepted in the pipeline meets this basic requirement prior to analysis.

FastQValidator [1] Version 1.0.5 is used to validate the file format. FastQValidator is a C++ program that checks that the four mandatory lines found in fastq format files are present in the input data file. It also ensures these lines are in the right format with unique read IDs and that there is similarity in the length of the raw sequence line and the quality string line.

The thresholds set for all the QC steps were adopted by consensus agreement at the ReSeqTB Expert panel meeting in Geneva Switzerland on the 27^th^ and 28^th^ of July 2015. A custom script embedded in the pipeline script estimates the average genome coverage. The QC threshold set for the acceptable minimum average genome coverage is 10X. This implies that for an input fastq file to be acceptable there should be an average of 10 sequence reads overlapping each nucleotide base position of the TB genome. The need for this minimum level of coverage is to establish prior confidence in the base calls from the sequencing platform before other downstream analysis.

The validated input data is assessed for species specificity using Kraken [3] Version 0.10.5. Kraken is a tool developed in C++ and Perl programing languages used to assign taxonomic labels to short sequence reads. Kraken details the percentage of sequence reads within each sample that map to particular taxonomic groupings. We set a threshold of 90% as the minimum acceptable proportion of sequence reads in the input data that has to map to the MTBC group of Kraken’s database.

If the file passes the Kraken threshold, we assess the raw sequence data for initial QC using the Prinseq-lite.pl [2] Version 0.20.4 tool and the FastQC tool Version 0.11.5.

- 1. **Sequence reads mapping & refinement**

The MTB genome H37Rv (NCBI Accession: NC_000962.3) is the reference genome used for mapping sequencing reads in the pipeline. Sequence reads are mapped to the reference genome using the Burrows Wheeler Alignment (BWA) [4] Version 0.7.12. BWA is a C++ bioinformatics tool designed for mapping relatively low divergent sequences against increasingly large reference genomes. The Maximal Exact Matches (MEM) algorithm of BWA, which is fast and suitable for the long Illumina sequence reads, was implemented for the mapping in the pipeline using default settings.

The quality of the mapping is reported using the Mapping quality QC Tool, Qualimap [5] Version 2.1.1. Qualimap outputs detailed reports on the sequence alignment using the ‘bamqc’ option of the tool. The report includes the mapping quality of the alignments, the genome coverage, nucleotide distribution and the mean and median insert size. Qualimap also outputs visualization files that could highlight any problems with the mapping process.

Next, the mapped data is refined to eliminate similar sequence reads using the bioinformatics tool suite Picard Tools [6] Version 1.134. This is achieved using the ‘MarkDuplicates’ options of the tool. Subsequently, the reads are also re-aligned proximal to insertions and deletions (Indels) to compensate for the stricter calling of gaps by mappers. The Genome Analysis Toolkit (GATK) [7] Version 3.4.0 is applied to make this re-alignment, using the ‘IndelRealigner’ option within GATK. The ‘BaseRecalibrator’ option of GATK is then used to recalculate base quality scores across the genome of isolates using machine-learning approaches to correct for error rates. Both Picard Tools and GATK are implemented in the JAVA programming platform.

- 1. **Variant Calling**

Variants are called using GATK ‘and Samtools [9] Version 1.2, both are C++ program that includes a suite of bioinformatics tools used to process and analyze NGS data. The UnifiedGenotyper’ and the ‘mpileup’ command are respectively used to generate variant and genotype calls from the preprocessed genomic data. The QC Thresholds supplied to this command include Q20 Minimum base quality, Q20 Minimum mapping quality, 10X Minimum Read depth and a maximum 3 SNPs within any 10 nucleotide base region for SNP clusters. This implies a minimum 99% accuracy in calling genetic variants, at the minimum of 10 sequence read coverage for each base position. This threshold also eliminates SNPs that occur in clusters within a short span of nucleotide bases, which are often spurious and artefacts of the sequencing platform. The output is represented in a variant call format (VCF) file, filtered to remove repeat and problematic loci in the *M. tuberculosis* genome (see Appendix 1). The VCF file contains information on all the variant positions, the reference and alternate base positions as well as details on the quality and genotyping information for each sample.

- 1. **Variant Annotation & Lineage Analysis**

The VCF file serves as an input file for the Functional Annotation Tool SnpEff [10] Version 4.1. SnpEff annotates each variant position in the VCF file using the same reference genome used to map the sequence reads in the first place. We built the H37Rv reference annotation file used for functional annotation into the application’s database using the GenBank file for NC_000962.3 and following the developer’s instruction for creating custom databases within the tool.

The custom python script parse_annotation.py reformats SnpEff output to a format easily mapped to the ReSeqTB database.

The custom python script lineage_parser.py performs a lineage analysis, to determine the lineage and sub-lineage of each analyzed strain. The informative SNPs which this script uses to make the lineage inferences is based on a set of 62 diagnostic SNPs defined by Coll *et al* [8].

| **Sample ID** | **Gene** | **Original**  **Position** | **Deletion**  **Size** | **UVP inferred Position** | **UVP deletion**  **size** |
| --- | --- | --- | --- | --- | --- |
| M_africanum_0_1bp_deletionMAF_20580 | MAF_20580 | 2281578 | 1 | 2281577 | 1 |
| M_africanum_1_1bp_deletionMAF_20580 | MAF_20580 | 2282046 | 1 | 2282046 | 1 |
| M_africanum_2_1bp_deletionMAF_20580 | MAF_20580 | 2281716 | 1 | 2281716 | 1 |
| M_africanum_3_1bp_deletionMAF_20580 | MAF_20580 | 2281944 | 1 | 2281943 | 1 |
| M_africanum_4_1bp_deletionMAF_20580 | MAF_20580 | 2281677 | 1 | 2281677 | 1 |
| M_africanum_0_2-5bp_deletionMAF_20580 | MAF_20580 | 2281925 | 2 | 2281925 | 2 |
| M_africanum_1_2-5bp_deletionMAF_20580 | MAF_20580 | 2281567 | 2 | 2281566 | 2 |
| M_africanum_3_2-5bp_deletionMAF_20580 | MAF_20580 | 2281995 | 2 | 2281994 | 2 |
| M_africanum_2_2-5bp_deletionMAF_20580 | MAF_20580 | 2281524 | 3 | 2281524 | 3 |
| M_africanum_4_2-5bp_deletionMAF_20580 | MAF_20580 | 2281878 | 4 | 2281878 | 4 |
| M_africanum_1_6-10bp_deletionMAF_20580 | MAF_20580 | 2281754 | 6 | 2281754 | 6 |
| M_africanum_3_6-10bp_deletionMAF_20580 | MAF_20580 | 2281949 | 7 | 2281948 | 7 |
| M_africanum_4_6-10bp_deletionMAF_20580 | MAF_20580 | 2281567 | 7 | 2281567 | 7 |
| M_africanum_2_6-10bp_deletionMAF_20580 | MAF_20580 | 2281934 | 8 | 2281932 | 8 |
| M_africanum_0_6-10bp_deletionMAF_20580 | MAF_20580 | 2281943 | 9 | 2281943 | 9 |
| M_africanum_4_10-20bp_deletionMAF_20580 | MAF_20580 | 2281554 | 11 | 2281553 | 11 |
| M_africanum_3_10-20bp_deletionMAF_20580 | MAF_20580 | 2281845 | 13 | 2281845 | 13 |
| M_africanum_2_10-20bp_deletionMAF_20580 | MAF_20580 | 2281799 | 14 | 2281798 | 14 |
| M_africanum_0_10-20bp_deletionMAF_20580 | MAF_20580 | 2281982 | 15 | 2281982 | 15 |
| M_africanum_1_10-20bp_deletionMAF_20580 | MAF_20580 | 2281612 | 17 | 2281612 | 17 |
| M_africanum_1_20-50bp_deletionMAF_20580 | MAF_20580 | 2282036 | 21 | 2282036 | 21 |
| M_africanum_0_20-50bp_deletionMAF_20580 | MAF_20580 | 2281918 | 27 | 2281918 | 27 |
| M_africanum_3_20-50bp_deletionMAF_20580 | MAF_20580 | 2281843 | 29 | 2281843 | 29 |
| M_africanum_2_20-50bp_deletionMAF_20580 | MAF_20580 | 2281843 | 31 | 2281843 | 31 |
| M_africanum_4_20-50bp_deletionMAF_20580 | MAF_20580 | 2281533 | 32 | 2281532 | 32 |
| M_africanum_33bp_deletionMAF_20580 | MAF_20580 | 2282020 | 33 | 2282020 | 33 |
| M_africanum_36bp_deletionMAF_20580 | MAF_20580 | 2281533 | 36 | 2281532 | 36 |
| M_africanum_42bp_deletionMAF_20580 | MAF_20580 | 2281974 | 42 | 2281973 | 42 |
| M_africanum_47bp_deletionMAF_20580 | MAF_20580 | 2281542 | 47 | 2281541 | 47 |
| M_africanum_49bp_deletionMAF_20580 | MAF_20580 | 2281737 | 49 | 2281734 | 49 |
| M_africanum_50bp_deletionMAF_20580 | MAF_20580 | 2282001 | 50 | 2282000 | 50 |
| M_africanum_52bp_deletionMAF_20580 | MAF_20580 | 2281977 | 52 | 2281977 | 52 |
| M_africanum_4_50-100bp_deletionMAF_20580 | MAF_20580 | 2281936 | 53 | Not inferred | Not Inferred |
| M_africanum_53bp_deletionMAF_20580 | MAF_20580 | 2281935 | 53 | Not inferred | Not Inferred |
| M_africanum_2_50-100bp_deletionMAF_20580 | MAF_20580 | 2281878 | 56 | Not inferred | Not Inferred |
| M_africanum_3_50-100bp_deletionMAF_20580 | MAF_20580 | 2281954 | 64 | Not inferred | Not Inferred |
| M_africanum_1_50-100bp_deletionMAF_20580 | MAF_20580 | 2281632 | 79 | Not inferred | Not Inferred |
| M_africanum_0_50-100bp_deletionMAF_20580 | MAF_20580 | 2281787 | 99 | Not inferred | Not Inferred |

**Supplementary Table S1**. Simulation analysis results indicating detection of simulated insertions and deletions in test samples.

**References**

1. FastqValidator – Genome Analysis [http://genome.sph.umich.edu/wiki/FastQValidator]
2. Schmieder R and Edwards R: Quality control and preprocessing of metagenomic datasets. *Bioinformatics* 2011, 27:863-864
3. Wood DE, Salzberg SL: [Kraken: ultrafast metagenomic sequence classification using exact alignments](http://genomebiology.com/2014/15/3/R46). *Genome Biology* 2014, 15:R46
4. Li H, Aligning Sequence Reads Clone Sequences and Assembly Contigs with BWA-MEM. arXiv e-print, 2013
5. García-Alcalde, et al. Qualimap: evaluating next generation sequencing alignment data. Bioinformatics(2012) 28 (20): 2678-2679
6. Picard Tools – [http://broadinstitute.github.io/picard/]
7. A framework for variation discovery and genotyping using next-generation DNA sequencing data DePristo M, Banks E, Poplin R, Garimella K, Maguire J, Hartl C, Philippakis A, del Angel G, Rivas MA, Hanna M, McKenna A, Fennell T, Kernytsky A, Sivachenko A, Cibulskis K, Gabriel S, Altshuler D, Daly M, 2011 NATURE GENETICS 43:491-498
8. Coll F, McNerney R, Guerra-Assuncao J, Glynn J, Perdigao J, Viveiros M, Portugal I, Pain A, Martin N, Clark T. A robust SNP barcode for typing Mycobacterium tuberculosis complex strains; Nature Communications 2014 Sep 1, 5: 4814
9. Li H A statistical framework for SNP calling, mutation discovery, association mapping and population genetical parameter estimation from sequencing data. Bioinformatics. 2011 Nov 1; 27(21):2987-93. Epub 2011 Sep 8.
10. Cingolani P, Platts A, Wang le L, Coon M, Nguyen T, Wang L, Land SJ, Lu X, Ruden DM A program for annotating and predicting the effects of single nucleotide polymorphisms, SnpEff: SNPs in the genome of Drosophila melanogaster strain w1118; iso-2; iso-3. Fly (Austin). 2012 Apr-Jun;6(2):80-92

**Appendix**

**Table S1** List of Excluded loci

**Chrom ChromStart ChromEnd locus tag Comment**

NC_000962 33582 33794 Rv0031 remnant of A transposase

NC_000962 103710 104663 Rv0094c 50bp_duplicated

NC_000962 104805 105215 Rv0095c 50bp_duplicated

NC_000962 105324 106715 Rv0096 PPE family protein

NC_000962 131382 132872 Rv0109 PE-PGRS family protein

NC_000962 149533 150996 Rv0124 PE-PGRS family protein

NC_000962 177543 179309 Rv0151c PE family protein

NC_000962 179319 180896 Rv0152c PE family protein

NC_000962 187433 188839 Rv0159c PE family protein

NC_000962 188931 190439 Rv0160c PE family protein

NC_000962 307877 309547 Rv0256c PPE family protein

NC_000962 309699 310073 Rv0257 50bp_duplicated

NC_000962 332708 333136 Rv0277c 50bp_duplicated

NC_000962 333437 336310 Rv0278c PE-PGRS family protein

NC_000962 336560 339073 Rv0279c PE-PGRS family protein

NC_000962 339364 340974 Rv0280 PPE family protein

NC_000962 349624 349932 Rv0285 PE family protein

NC_000962 349935 351476 Rv0286 PPE family protein

NC_000962 361334 363109 Rv0297 PE-PGRS family protein

NC_000962 366150 372764 Rv0304c PPE family protein

NC_000962 372820 375711 Rv0305c PPE family protein

NC_000962 399535 400050 Rv0335c PE family protein

NC_000962 400192 401703 Rv0336 50bp_duplicated

NC_000962 423639 424019 Rv0353 50bp_duplicated

NC_000962 424269 424694 Rv0354c PPE family protein

NC_000962 424777 434679 Rv0355c PPE family protein

NC_000962 466672 467406 Rv0387c PPE family protein

NC_000962 467459 468001 Rv0388c PPE family protein

NC_000962 472781 474106 Rv0393 50bp_duplicated

NC_000962 475816 476184 Rv0397 50bp_duplicated

NC_000962 530751 532214 Rv0442c PPE family protein

NC_000962 543174 544730 Rv0453 PPE family protein

NC_000962 576787 577338 Rv0487 50bp_duplicated

NC_000962 579349 580581 Rv0490 50bp_duplicated

NC_000962 606551 608062 Rv0515 50bp_duplicated

NC_000962 622793 624577 Rv0532 PE-PGRS family protein

NC_000962 630040 631686 Rv0538 50bp_duplicated

NC_000962 671996 675916 Rv0578c PE-PGRS family protein

NC_000962 701406 702014 Rv0605 repeat region

NC_000962 701406 702014 Rv0605 resolvase

NC_000962 831776 832303 Rv0740 50bp_duplicated

NC_000962 832534 832848 Rv0741 transposase

NC_000962 832981 833508 Rv0742 PE-PGRS family protein

NC_000962 835701 838052 Rv0746 PE-PGRS family protein

NC_000962 838451 840856 Rv0747 PE-PGRS family protein

NC_000962 842033 842278 Rv0750 50bp_duplicated

NC_000962 846159 847913 Rv0754 PE-PGRS family protein

NC_000962 850527 850342 Rv0755A transposase

NC_000962 848103 850040 Rv0755c PPE family protein

NC_000962 889072 889398 Rv0795 transposase IS6110

NC_000962 889347 890333 Rv0796 transposase IS6110

NC_000962 890388 891482 Rv0797 50bp_duplicated

NC_000962 908181 908483 Rv0814c 50bp_duplicated

NC_000962 916477 917646 Rv0823c 50bp_duplicated

NC_000962 921575 921865 Rv0829 50bp_duplicated

NC_000962 924951 925364 Rv0832 PE-PGRS family protein

NC_000962 925361 927610 Rv0833 PE-PGRS family protein

NC_000962 927837 930485 Rv0834c PE-PGRS family protein

NC_000962 947312 947644 Rv0850 transposase

NC_000962 964312 965535 Rv0867c 50bp_duplicated

NC_000962 968424 970244 Rv0872c PE-PGRS family protein

NC_000962 976872 978203 Rv0878c PPE family protein

NC_000962 1020058 1021329 Rv0915c PPE family protein

NC_000962 1021344 1021643 Rv0916c PE family protein

NC_000962 1025497 1026816 Rv0920c transposase

NC_000962 1027104 1027685 Rv0921 resolvase

NC_000962 1027685 1029337 Rv0922 transposase

NC_000962 1090373 1093144 Rv0977 PE-PGRS family protein

NC_000962 1093361 1094356 Rv0978c PE-PGRS family protein

NC_000962 1095078 1096451 Rv0980c PE-PGRS family protein

NC_000962 1158918 1159307 Rv1034c transposase

NC_000962 1159375 1160061 Rv1035c transposase

NC_000962 1160095 1160433 Rv1036c truncated IS1560 transposase

NC_000962 1160544 1160828 Rv1037c 50bp_duplicated

NC_000962 1160855 1161151 Rv1038c 50bp_duplicated

NC_000962 1161297 1162472 Rv1039c PPE family protein

NC_000962 1162549 1163376 Rv1040c PE family protein

NC_000962 1164572 1165435 Rv1041c IS like-2 transposase

NC_000962 1165092 1165499 Rv1042c IS like-2 transposase

NC_000962 1169423 1170670 Rv1047 transposase

NC_000962 1188421 1190424 Rv1067c PE-PGRS family protein

NC_000962 1190757 1192148 Rv1068c PE-PGRS family protein

NC_000962 1211560 1213863 Rv1087 PE-PGRS family protein

NC_000962 1214513 1214947 Rv1088 PE family protein

NC_000962 1214769 1215131 Rv1089 PE family protein

NC_000962 1216469 1219030 Rv1091 PE-PGRS family protein

NC_000962 1251617 1252972 Rv1128c repeat_region

NC_000962 1262272 1264128 Rv1135c PPE family protein

NC_000962 1276300 1277748 Rv1148c 50bp_duplicated

NC_000962 1277893 1278300 Rv1149 transposase

NC_000962 1278269 1278820 Rv1150 Possible fragment of transposase

NC_000962 1298764 1299804 Rv1168c PPE family protein

NC_000962 1299822 1300124 Rv1169c PE family protein

NC_000962 1301755 1302681 Rv1172c PE family protein

NC_000962 1339003 1339302 Rv1195 PE family protein

NC_000962 1339349 1340524 Rv1196 PPE family protein

NC_000962 1340659 1340955 Rv1197 50bp_duplicated

NC_000962 1341006 1341290 Rv1198 50bp_duplicated

NC_000962 1341358 1342605 Rv1199c transposase

NC_000962 1357293 1357625 Rv1214c PE family protein

NC_000962 1384989 1386677 Rv1243c PE-PGRS family protein

NC_000962 1441348 1442718 Rv1288 50bp_duplicated

NC_000962 1450697 1451779 Rv1295 50bp_duplicated

NC_000962 1468171 1469505 Rv1313c transposase

NC_000962 1479199 1480824 Rv1318c 50bp_duplicated

NC_000962 1480894 1482501 Rv1319c 50bp_duplicated

NC_000962 1488154 1489965 Rv1325c PE-PGRS family protein

NC_000962 1532443 1533633 Rv1361c PPE family protein

NC_000962 1541994 1542980 Rv1369c transposase

NC_000962 1542929 1543255 Rv1370c transposase

NC_000962 1561464 1561772 Rv1386 PE family protein

NC_000962 1561769 1563388 Rv1387 PPE family protein

NC_000962 1572127 1573857 Rv1396c PE-PGRS family protein

NC_000962 1606386 1607972 Rv1430 PE family protein

NC_000962 1618209 1619684 Rv1441c PE-PGRS family protein

NC_000962 1630638 1634627 Rv1450c PE-PGRS family protein

NC_000962 1636004 1638229 Rv1452c PE-PGRS family protein

NC_000962 1643319 1644260 Rv1458c 50bp_duplicated

NC_000962 1655609 1656721 Rv1468c PE-PGRS family protein

NC_000962 1678942 1679172 Rv1489A 50bp_duplicated

NC_000962 1684005 1686257 Rv1493 50bp_duplicated

NC_000962 1751297 1753333 Rv1548c PPE family protein

NC_000962 1761744 1762937 Rv1557 repeat_region

NC_000962 1762947 1763393 Rv1558 repeat_region

NC_000962 1779194 1779298 Rv1572c repeat_region

NC_000962 1779930 1780241 Rv1574 repeat_region

NC_000962 1780199 1780699 Rv1575 repeat_region

NC_000962 1779314 1779724 Rv1573 phiRV1 phage protein

NC_000962 1779930 1780241 Rv1574 phiRV1 phage related protein

NC_000962 1780199 1780699 Rv1575 phiRV1 phage protein

NC_000962 1780643 1782064 Rv1576c phiRV1 phage protein

NC_000962 1782072 1782584 Rv1577c phiRv1 phage protein

NC_000962 1782758 1783228 Rv1578c phiRv1 phage protein

NC_000962 1783309 1783623 Rv1579c phiRv1 phage protein

NC_000962 1783620 1783892 Rv1580c phiRv1 phage protein

NC_000962 1783906 1784301 Rv1581c phiRv1 phage protein

NC_000962 1784497 1785912 Rv1582c phiRv1 phage protein

NC_000962 1785912 1786310 Rv1583c phiRv1 phage protein

NC_000962 1786307 1786528 Rv1584c phiRv1 phage protein

NC_000962 1786584 1787099 Rv1585c phiRv1 phage protein

NC_000962 1787096 1788505 Rv1586c phiRv1 integrase

NC_000962 1788162 1789163 Rv1587c REP13E12 repeat-containing protein

NC_000962 1789168 1789836 Rv1588c REP13E12 repeat-containing protein

NC_000962 1855764 1856696 Rv1646 PE family protein

NC_000962 1862347 1865382 Rv1651c PE-PGRS family protein

NC_000962 1927211 1928575 Rv1702c repeat_region

NC_000962 1931497 1932654 Rv1705c PPE family protein

NC_000962 1932694 1933878 Rv1706c PPE family protein

NC_000962 1981614 1984775 Rv1753c PPE family protein

NC_000962 1987745 1988731 Rv1756c putative transposase

NC_000962 1988680 1989006 Rv1757c putative transposase

NC_000962 1989042 1989566 Rv1758 putative transposase

NC_000962 1989833 1992577 Rv1759c PE-PGRS family protein

NC_000962 1996152 1996478 Rv1763 putative transposase

NC_000962 1996427 1997413 Rv1764 putative transposase

NC_000962 1999357 1999142 Rv1765A transposase

NC_000962 1997418 1998515 Rv1765c 50bp_duplicated

NC_000962 2000614 2002470 Rv1768 PE-PGRS family protein

NC_000962 2025301 2026398 Rv1787 PPE family protein

NC_000962 2026477 2026776 Rv1788 PE family protein

NC_000962 2026790 2027971 Rv1789 PPE family protein

NC_000962 2028425 2029477 Rv1790 PPE family protein

NC_000962 2029904 2030203 Rv1791 PE family protein

NC_000962 2030694 2030978 Rv1793 50bp_duplicated

NC_000962 2039453 2041420 Rv1800 PPE family protein

NC_000962 2042001 2043272 Rv1801 PPE family protein

NC_000962 2043384 2044775 Rv1802 PPE family protein

NC_000962 2044923 2046842 Rv1803c PE-PGRS family protein

NC_000962 2048072 2048371 Rv1806 PE family protein

NC_000962 2048398 2049597 Rv1807 PPE family protein

NC_000962 2049921 2051150 Rv1808 PPE family protein

NC_000962 2051282 2052688 Rv1809 PPE family protein

NC_000962 2061178 2062674 Rv1818c PE-PGRS family protein

NC_000962 2073943 2074437 Rv1829 50bp_duplicated

NC_000962 2087971 2089518 Rv1840c PE-PGRS family protein

NC_000962 2156706 2157299 Rv1910c 50bp_duplicated

NC_000962 2157382 2157987 Rv1911c 50bp_duplicated

NC_000962 2162932 2167311 Rv1917c PPE family protein

NC_000962 2167649 2170612 Rv1918c PPE family protein

NC_000962 2195989 2197353 Rv1945 repeat_region

NC_000962 2226244 2227920 Rv1983 PE-PGRS family protein

NC_000962 2260665 2261144 Rv2013 transposase

NC_000962 2261098 2261688 Rv2014 transposase

NC_000962 2261816 2263072 Rv2015c 50bp_duplicated

NC_000962 2294531 2306986 Rv2048c 50bp_duplicated

NC_000962 2338709 2340874 Rv2082 50bp_duplicated

NC_000962 2343027 2343332 Rv2085 repeat_region

NC_000962 2347373 2348554 Rv2090 50bp_duplicated

NC_000962 2365465 2365791 Rv2105 transposase

NC_000962 2365740 2366726 Rv2106 transposase

NC_000962 2367359 2367655 Rv2107 PE family protein

NC_000962 2367711 2368442 Rv2108 PPE family protein

NC_000962 2370905 2372569 Rv2112c 50bp_duplicated

NC_000962 2381071 2382492 Rv2123 PPE family protein

NC_000962 2387202 2387972 Rv2126c PE-PGRS family protein

NC_000962 2423240 2424838 Rv2162c PE-PGRS family protein

NC_000962 2430159 2431145 Rv2167c transposase

NC_000962 2431094 2431420 Rv2168c transposase

NC_000962 2439282 2439947 Rv2177c transposase

NC_000962 2459678 2461327 Rv2196 50bp_duplicated

NC_000962 2530836 2531897 Rv2258c 50bp_duplicated

NC_000962 2549124 2550029 Rv2277c 50bp_duplicated

NC_000962 2550065 2550391 Rv2278 transposase

NC_000962 2550340 2551326 Rv2279 transposase

NC_000962 2600731 2601879 Rv2328 PE family protein

NC_000962 2617667 2618908 Rv2340c PE-PGRS family protein

NC_000962 2625888 2626172 Rv2346c 50bp_duplicated

NC_000962 2626223 2626519 Rv2347c 50bp_duplicated

NC_000962 2632923 2634098 Rv2352c PPE family protein

NC_000962 2634528 2635592 Rv2353c PPE family protein

NC_000962 2635628 2635954 Rv2354 transposase

NC_000962 2635903 2636889 Rv2355 transposase

NC_000962 2637688 2639535 Rv2356c PPE family protein

NC_000962 2651753 2651938 Rv2371 PE-PGRS family protein

NC_000962 2692799 2693884 Rv2396 PE-PGRS family protein

NC_000962 2706017 2706736 Rv2408 PE family protein

NC_000962 2720776 2721777 Rv2424c transposase

NC_000962 2727336 2727920 Rv2430c PPE family protein

NC_000962 2727967 2728266 Rv2431c PE family protein

NC_000962 2762531 2763175 Rv2460c repeat_region

NC_000962 2763172 2763774 Rv2461c repeat_region

NC_000962 2784657 2785643 Rv2479c transposase

NC_000962 2785592 2785918 Rv2480c transposase

NC_000962 2795301 2797385 Rv2487c PE-PGRS family protein

NC_000962 2800846 2801145 Rv2489c repeat_region

NC_000962 2801254 2806236 Rv2490c PE-PGRS family protein

NC_000962 2828556 2829803 Rv2512c IS1081 transposase

NC_000962 2835785 2837263 Rv2519 PE family protein

NC_000962 2866468 2867127 Rv2543 50bp_duplicated

NC_000962 2867124 2867786 Rv2544 50bp_duplicated

NC_000962 2921551 2923182 Rv2591 PE-PGRS family protein

NC_000962 2935046 2936788 Rv2608 PPE family protein

NC_000962 2943600 2944985 Rv2615c PE-PGRS family protein

NC_000962 2960105 2962441 Rv2634c PE-PGRS family protein

NC_000962 2972160 2972486 Rv2648 transposase IS6110

NC_000962 2972435 2973421 Rv2649 transposase IS6110

NC_000962 2973795 2975234 Rv2650c phiRv2 prophage protein

NC_000962 2975242 2975775 Rv2651c phiRv2 prophage protease

NC_000962 2975928 2976554 Rv2652c phiRv2 prophage protein

NC_000962 2976586 2976909 Rv2653c phiRv2 prophage protein

NC_000962 2976989 2977234 Rv2654c phiRv2 prophage protein

NC_000962 2977231 2978658 Rv2655c phiRv2 prophage protein

NC_000962 2978660 2979052 Rv2656c phiRv2 prophage protein

NC_000962 2979049 2979309 Rv2657c phiRv2 prophage protein

NC_000962 2979691 2980818 Rv2659c phiRv2 prophage integrase

NC_000962 2982699 2982980 Rv2665 50bp_duplicated

NC_000962 2983071 2983874 Rv2666 truncated IS1081 transposase

NC_000962 2989291 2990592 Rv2673 50bp_duplicated

NC_000962 2996105 2996737 Rv2680 50bp_duplicated

NC_000962 3005845 3007062 Rv2689c 50bp_duplicated

NC_000962 3007236 3009209 Rv2690c repeat_region

NC_000962 3053914 3055491 Rv2741 PE-PGRS family protein

NC_000962 3076894 3078078 Rv2768c PPE family protein

NC_000962 3078158 3078985 Rv2769c PE family protein

NC_000962 3079309 3080457 Rv2770c PPE family protein

NC_000962 3082352 3082756 Rv2774c 50bp_duplicated

NC_000962 3100202 3101581 Rv2791c transposase

NC_000962 3101581 3102162 Rv2792c resolvase

NC_000962 3112867 3113271 Rv2805 50bp_duplicated

NC_000962 3113658 3114812 Rv2807 50bp_duplicated

NC_000962 3115741 3116142 Rv2810c transposase

NC_000962 3116818 3118227 Rv2812 transposase

NC_000962 3120566 3121552 Rv2814c transposase

NC_000962 3121501 3121827 Rv2815c transposase

NC_000962 3132892 3133539 Rv2825c 50bp_duplicated

NC_000962 3135788 3136333 Rv2828c 50bp_duplicated

NC_000962 3162268 3164115 Rv2853 PE-PGRS family protein

NC_000962 3170720 3171646 Rv2859c 50bp_duplicated

NC_000962 3191644 3192201 Rv2882c 50bp_duplicated

NC_000962 3194166 3195548 Rv2885c transposase

NC_000962 3195545 3196432 Rv2886c resolvase

NC_000962 3200794 3202020 Rv2892c PPE family protein

NC_000962 3245445 3251075 Rv2931 50bp_duplicated

NC_000962 3251072 3255688 Rv2932 50bp_duplicated

NC_000962 3288464 3289705 Rv2943 IS1533 transposase

NC_000962 3289705 3290235 Rv2943A transposase

NC_000962 3289790 3290506 Rv2944 IS1533 transposase

NC_000962 3313283 3313672 Rv2961 transposase

NC_000962 3332787 3333788 Rv2977c 50bp_duplicated

NC_000962 3333785 3335164 Rv2978c transposase

NC_000962 3335164 3335748 Rv2979c resolvase

NC_000962 3335960 3336505 Rv2980 50bp_duplicated

NC_000962 3378415 3378329 Rv3018A PE family protein

NC_000962 3376939 3378243 Rv3018c PPE family protein

NC_000962 3379376 3380452 Rv3021c PPE family protein

NC_000962 3380993 3380679 Rv3022A PE family protein

NC_000962 3380440 3380682 Rv3022c PPE family protein

NC_000962 3381375 3382622 Rv3023c transposase

NC_000962 3481451 3482698 Rv3115 transposase

NC_000962 3490476 3491651 Rv3125c PPE family protein

NC_000962 3501334 3501732 Rv3135 PPE family protein

NC_000962 3501794 3502936 Rv3136 PPE family protein

NC_000962 3510088 3511317 Rv3144c PPE family protein

NC_000962 3527391 3529163 Rv3159c PPE family protein

NC_000962 3551281 3551607 Rv3184 transposase

NC_000962 3551556 3552542 Rv3185 transposase

NC_000962 3552764 3553090 Rv3186 transposase

NC_000962 3553039 3554025 Rv3187 transposase

NC_000962 3557311 3558345 Rv3191c transposase

NC_000962 3663689 3664222 Rv3281 50bp_duplicated

NC_000962 3710433 3710759 Rv3325 transposase

NC_000962 3710708 3711694 Rv3326 transposase

NC_000962 3711749 3713461 Rv3327 transposase

NC_000962 3729364 3736935 Rv3343c PPE family protein

NC_000962 3736984 3738438 Rv3344c PE-PGRS family protein

NC_000962 3738158 3742774 Rv3345c PE-PGRS family protein

NC_000962 3743198 3743455 Rv3346c 50bp_duplicated

NC_000962 3743711 3753184 Rv3347c PPE family protein

NC_000962 3753765 3754256 Rv3348 transposase

NC_000962 3754293 3755033 Rv3349c transposase

NC_000962 3755952 3767102 Rv3350c PPE family protein

NC_000962 3769514 3769807 Rv3355c 50bp_duplicated

NC_000962 3778568 3780334 Rv3367 PE-PGRS family protein

NC_000962 3795100 3796086 Rv3380c transposase

NC_000962 3796035 3796361 Rv3381c transposase

NC_000962 3800092 3800796 Rv3386 transposase

NC_000962 3800786 3801463 Rv3387 transposase

NC_000962 3801653 3803848 Rv3388 PE-PGRS family protein

NC_000962 3841714 3842076 Rv3424c 50bp_duplicated

NC_000962 3842239 3842769 Rv3425 PPE family protein

NC_000962 3843036 3843734 Rv3426 PPE family protein

NC_000962 3843885 3844640 Rv3427c transposase

NC_000962 3844738 3845970 Rv3428c transposase

NC_000962 3847165 3847701 Rv3429 PPE family protein

NC_000962 3847642 3848805 Rv3430c transposase

NC_000962 3849294 3850139 Rv3431c repeat region

NC_000962 3883525 3884193 Rv3466 repeat region

NC_000962 3883964 3884917 Rv3467 repeat region

NC_000962 3890830 3891156 Rv3474 transposase IS6110

NC_000962 3891105 3892091 Rv3475 transposase IS6110

NC_000962 3894093 3894389 Rv3477 PE family protein

NC_000962 3894426 3895607 Rv3478 PE family protein

NC_000962 3926569 3930714 Rv3507 PE-PGRS family protein

NC_000962 3931005 3936710 Rv3508 PE-PGRS family protein

NC_000962 3939617 3941761 Rv3511 PE-PGRS family protein

NC_000962 3941724 3944963 Rv3512 PE-PGRS family protein

NC_000962 3945092 3945748 Rv3513c 50bp_duplicated

NC_000962 3945794 3950263 Rv3514 PE-PGRS family protein

NC_000962 3950824 3952470 Rv3515c 50bp_duplicated

NC_000962 3969343 3970563 Rv3532 PPE family protein

NC_000962 3970705 3972453 Rv3533c PPE family protein

NC_000962 3978059 3979498 Rv3539 PPE family protein

NC_000962 3997980 3999638 Rv3558 PPE family protein

NC_000962 4031404 4033158 Rv3590c PE-PGRS family protein

NC_000962 4036731 4038050 Rv3595c PE-PGRS family protein

NC_000962 4052950 4053603 Rv3611 50bp_duplicated

NC_000962 4059984 4060268 Rv3619c 50bp_duplicated

NC_000962 4060295 4060591 Rv3620c 50bp_duplicated

NC_000962 4060648 4061889 Rv3621c PPE family protein

NC_000962 4061899 4062198 Rv3622c PE family protein

NC_000962 4075752 4076099 Rv3636 transposase

NC_000962 4076484 4076984 Rv3637 transposase

NC_000962 4076984 4077730 Rv3638 transposase

NC_000962 4077884 4078450 Rv3639c 50bp_duplicated

NC_000962 4078520 4079749 Rv3640c transposase

NC_000962 4091233 4091517 Rv3650 PE family protein

NC_000962 4119795 4120955 Rv3680 50bp_duplicated

NC_000962 4153740 4155674 Rv3710 50bp_duplicated

NC_000962 4189285 4190232 Rv3738c PPE family protein

NC_000962 4190284 4190517 Rv3739c PPE family protein

NC_000962 4196171 4196506 Rv3746c PE family protein

NC_000962 4252993 4254327 Rv3798 transposase

NC_000962 4276571 4278085 Rv3812 PE-PGRS family protein

NC_000962 4299812 4301566 Rv3826 50bp_duplicated

NC_000962 4301563 4302789 Rv3827c transposase

NC_000962 4302786 4303397 Rv3828c resolvase

NC_000962 4318775 4319266 Rv3844 transposase

NC_000962 4351075 4352181 Rv3873 PPE family protein

NC_000962 4353010 4355010 Rv3876 50bp_duplicated

NC_000962 4374484 4375683 Rv3892c PPE family protein

NC_000962 4375762 4375995 Rv3893c PE family protein

NC_000962 1306002 1306201 IG1195_Rv1174c-Rv1175c

NC_000962 154130 154231 IG127_Rv0126-Rv0127

NC_000962 1907321 1907593 IG1711_Rv1682-Rv1683

NC_000962 23182 23269 IG18_Rv0018c-Rv0019c

NC_000962 3318816 3318900 IG3012_Rv2965c-Rv2966c

NC_000962 3319468 3319662 IG3013_Rv2966c-Rv2967c

NC_000962 616832 616845 IG533_Rv0525-Rv0526

NC_000962 642812 642888 IG559_Rv0551c-Rv0552

NC_000962 706930 706947 IG622_Rv0612-Rv0613c

NC_000962 80194 80623 IG71_Rv0071-Rv0072

NC_000962 863159 863255 IG784_Rv0769-Rv0770

NC_000962 960152 960341 IG877_Rv0861c-Rv0862c
